# Bread as a Valuable Raw Material in Craft Ale Beer Brewing

**DOI:** 10.3390/foods11193013

**Published:** 2022-09-28

**Authors:** Carlos Martin-Lobera, Fernando Aranda, Patricia Lozano-Martinez, Isabel Caballero, Carlos A. Blanco

**Affiliations:** 1 Department of Agricultural and Forestry Engineering (Food Technology Area), E.T.S. Agricultural Engineering, University of Valladolid, 34004 Palencia, Spain; 2 Agri-Food Division and Sustainable Processes, CARTTIF Foundation, 47151 Boecillo, Spain

**Keywords:** brew, beer, surplus bread, antioxidants, polyphenols, organoleptic properties, volatile composition

## Abstract

One of the ingredients used for brewing is barley, which should be malted; it is considered the most polluting agricultural input. On the other hand, food wastage is today a widespread problem that causes significant environmental damage and also generates large economic losses worldwide. One of the most wasted food products is bread; it is estimated that hundreds of tons of bread are wasted every day worldwide. In this study, the brewing of ale beers with bread was carried out. For this purpose, up to 50% of the malt weight was replaced by different types of bread: wheat bread, whole wheat bread, rye bread, and corn bread. A physicochemical and sensory comparison was made with 100% malt ale beer. All beers brewed with bread had an alcoholic strength similar to that of the control beer, except the corn beer. Beers brewed with whole grain bread showed a higher antioxidant capacity and a higher total polyphenol content. The sensory analysis presented different profiles depending on the type of bread; in general, the addition of bread created a greater olfactory intensity in nose. Thus, it was found that it is possible to brew beer with bread substituting up to 50% of the malt. In addition, it was also shown that the beer brewed with whole wheat bread had similar characteristics to the control beer, even improving some beneficial health properties, representing a great advantage for the brewing industry all over the world.

## 1. Introduction

Beer is a classic alcoholic beverage, and it is one of the most internationally popular drinks [1]. It is a beverage usually made from malted cereal grain (such as barley), yeast, hops, and water. Sometimes, adjuncts and food additives are also included. There are different types of beer, each of which has specific organoleptic properties encompassing gustative, visual, and aromatic perceptions. These properties are affected by the raw materials, the fermentation of the wort, and the technological conditions used in production and packaging [1,2].

Concern about environmental issues and the impact that companies have on them has increased. This also affects beer consumers, creating new challenges for the brewing industry. Problems related to water consumption, energy efficiency, waste generation, emissions management, and the environmental impact of the brewing process have become important topics of discussion and are receiving increased attention from large and small breweries [3].

The beer brewing process has several outputs, such as brewer’s spent grain, hot trub, and residual brewer’s yeast, which may cause negative environmental effects [4]. Producing and selling beer requires various types of inputs, such as raw materials, machinery, packaging materials, and transportation [3]. These impacts differ depending on the stage of the beer product life cycle. The total process can be divided into five different stages: the production and transportation of raw materials, beer production, wastewater treatment in the brewery, the production and transportation of packaging, and the distribution of the final product to customers [5]. The production and transport of these ingredients have been identified as key contributors to the life cycle environmental impact of beer [6,7].

Food waste generates substantial economic losses globally [8], and bread, in particular, is the most commonly wasted food product in developed countries.

The production of bread is estimated to generate about 100 million tons per year, 65% of which is consumed in Europe [9]. During the storage of bread, a complex physicochemical process defined as staling occurs, mainly driven by the loss of moisture and retrogradation of starch [10]. Moreover, bread composition makes it susceptible to microbial attack, which is why preservatives that inhibit spore, mold, and/or yeast growth are used to reduce spoilage and ensure safety [11]. Determining the precise amount of bread wasted during its life cycle is a difficult task, but it is estimated that hundreds of tons are wasted daily worldwide [9]. However, the limited shelf-life is only one of the reasons why this large amount of bread is wasted.

Bread is a starchy food and an important source of easily extractable fermentable sugars, which is in direct contrast to lignocellulosic feedstocks, where harsh physical, chemical, and/or enzymatic pre-treatment processes are required for the release of fermentable sugars. During the last decade, several initiatives focused on finding alternatives for recycling bread waste have taken place: bread has been proposed as a substrate to produce chemical products for pharmaceutical companies, the food industry, biofuels, and enzymes [12,13,14]; as a substrate for the biomass production of *Saccharomyces cerevisiae* [15]; and in the production of ingredients for food processing [16]. Bread residues contain a high concentration of starch (over 70% dry matter) and protein (up to 14% dry matter) [9], and treatment with amylases, amyloglucosidases, and proteases easily leads to the release of available compounds for microbial growth [17].

Craft beer is characterized by the use of high-quality ingredients together with non-traditional ones (which, apart from reducing costs, adds special flavor and sensorial characteristics), as shown in our previous article [18].

Recently, some small breweries have started to use surplus bread to make their beers, replacing part of the malted barley that was originally used as a source of sugar for fermentation [19,20]. Studies already carried out have shown that the proportion of malt that can be replaced is limited [8], as malt contains enzymes necessary to break down bread starch into fermentable sugars, meaning that it is not practical to replace more than 25% of the malt. In this work, an attempt is made to valorize and recycle bread waste and brew ale beers using different types of bread as a partial substitute for malt. For this purpose, ale beer was brewed by replacing up to 50% of the malt weight with different types of bread: wheat bread, whole wheat bread, rye bread, and corn bread. Subsequently, a physicochemical comparison (including antioxidant activity and total polyphenol content) with 100% malt ale beer was made. A sensory comparison (visual and taste) between the different beers brewed was also developed.

## 2. Materials and Methods

### 2.1. Raw Materials

The brewing ingredients used for craft beer elaboration are shown in Table 1:

The yeast strain SafAle S-04 was used for the fermentation process in tanks, and the SafAle F-2 strain for fermentation in bottles; bread was purchased from La Tahona de Sahagún (Palencia, Spain), a local bread producer.

### 2.2. Reagents and Chemicals

2,2-Diphenyl-1-picrylhydrazyl (DPPH) was acquired from Sigma-Aldrich Química S.A., Madrid, Spain. Gallic acid, methanol, and Folin–Ciocalteu reagent were acquired from Merck Millipore, Madrid, Spain. Sodium hydroxide (NaOH) 0.01 N, sodium chloride (NaCl), D(+)-glucose anhydrous for ACS analysis, Coomassie blue G.250 (CBBG), and Bradford reagent were acquired from Panreac, Castellar del Vallés, Spain. All solutions were prepared using analytical grade reagents and distilled water.

### 2.3. Pale Ale Beer Production in the Pilot Brewery

A control pale ale beer was brewed using the malts and hops that were most frequently repeated in the consulted recipes, i.e., those for American pale ale, one of the most-consumed types [21]. The total amount of malt used was 1046 g per brew; in detail, the following amounts of malt were added to 6 L of mineral water: Pilsen (45% by weight), Munich type I (40% by weight), Biscuit (10% by weight), Cara Rye (5% by weight), mineral water, pelleted hops (Centennial, Cascade, and Simcoe), and yeast.

Also, four brews were made according to the control pale ale beer recipe, using different kinds of bread (wheat, rye, whole wheat, and corn) to replace, in each case, 50% of the malt weight with the same amount of stale bread weight.

The pale ale beers produced were named using abbreviations, as follows: control (ALE); wheat bread (WHIB); rye bread (RYEB); whole wheat bread, also known as brown wheat (BROB); and corn bread (CORB). All elaborations were brewed in duplicate, obtaining 5 L of beer in each case and labeling each one by the abbreviation previously mentioned, followed by the number 1 or 2 depending on the duplicate.

The malt and bread were milled at the University of Valladolid cellar (University of Valladolid Campus, Palencia, Spain) just before mashing. The resulting ground malt was mixed with preheated mineral water at 40 ± 1 °C for 20 min for mashing into a stainless macerator tank. In all cases, mashing took two hours, at 67 ± 1 °C with manual stirring, to convert the starches from the malt and bread into sugar. Finally, the mash temperature was raised to 78 ± 1 °C for a 10 min period, at a rate of 1 °C per min (mash out), to deactivate the enzymes; then, the mixtures remained at room temperature for 24 h.

Mashing was followed by wort separation and sparging until the desired amount of wort was collected. The sparging was performed with natural mineral water at 80 ± 1 °C until the final volume of 5 L was completed, in order to achieve a greater extraction of fermentable sugars.

For the lupulization step, wort boiling at 100 °C required 60 min, following the time scheme shown in Table 2, and resulted in a total bitterness of 30 IBU (international bitterness units):

All wort was fermented using the commercial yeast SafAle S-04, at 21 ± 1 °C, in 6 L tanks for 10 days. After beers reached the final attenuation degree, the temperature was gradually reduced to 4 °C in a cooling chamber; the beer remained for a week in the tanks to facilitate lees removal and beer maturation.

After the removal of lees, all the containers were tempered to 21 °C, and the carbonation phase was performed with 33 mL volume glass bottles, using dextrose and SafAle F-2 yeast, to obtain 2.0 bar of internal CO_2_ pressure. All bottles rested for 14 days to finish fermentation at 21 ± 1 °C.

Finally, beer bottles matured in a refrigerated chamber at 4 °C for 2 weeks.

### 2.4. Physicochemical Analysis

All analyses were performed in triplicate. Unfiltered samples were extracted, excepting the color method, in which samples were previously centrifugated in a centrifuge (Bunsen, model KOCH 1460, Humanes de Madrid, Spain) at 4000 rpm for 5 min, as well as filtered by a vacuum filter (model: Kitasato) and Millipore filters (Merck Millipore, Darmstadt, Germany) of 0.45 microns.

- Turbidity
- Beer turbidity was measured with a turbidimeter (Hanna Instruments, HI 98703 model, Eibar, Spain), and each sample was placed in a transparent glass container with a lid. Each of these containers was placed in the turbidimeter to obtain turbidity values in NTU (nephelometric turbidity units).
- pH: pH was measured with a pH-meter (HACH-LANGE, calibrated sensiON™ + pH3 model, Hospitalet, Spain).
- Acidity: pH-meter measurements were taken in continuous function. An acid–base titration was performed until a pH of 7 was reached. The results were expressed in terms of lactic acid percentage.
- Alcohol By Volume (ABV): an ebulliometer (GAB system, 1010006 model, Moja, Spain) was used. It was calibrated with a standard (distilled water). The boiling temperatures of the standard (water) and the test sample (beer) were compared, and the volumetric alcohol content was calculated with a precision of 0.1 ABV using a ruler scale.
- Color (EBC): color was measured on a spectrophotometer (ThermoFisher Scientific, model 20 Genesys UV-Vis, Madrid, Spain). A beer sample of 3 mL, previously filtered, was introduced into a standard glass cuvette of 1 cm. The absorbance at 430 nm was measured, and distilled water was used as a blank. The obtained value was transformed to the European Brewery Convention (EBC) scale, multiplying the value by 25.
- Dry Extract: dry extract was measured with a thermobalance (Gibertini Eurotherm brand, Novate Milanese, Italy). An identical weight of each beer sample (1 g) was placed on the balance. The water contained in the sample was evaporated, and the remaining solid (dry extract) was weighed. The percentage of dry extract can be obtained directly via the difference with the total sample introduced.

### 2.5. Total Polyphenol Content and Antioxidant Capacity in Craft Beers

Samples were previously centrifugated in a centrifuge (Bunsen, model KOCH 1460, Humanes de Madrid, Spain) at 4000 rpm for 5 min, as well as filtered by a vacuum filter (model: Kitasato) and Millipore filters (Merck Millipore, Madrid, Spain) of 0.45 microns.

- Total polyphenol content (TPC).
- The total polyphenol content was determined by the Folin–Ciocalteu method by measuring absorbance at 760 nm [22] using the spectrophotometer mentioned above. A calibration line was performed using different concentrations (0.0–30 mg/L) of standard solutions of gallic acid, resulting in the following equation: Y = 0.0243x + 0.0209, R = 0.9959. The concentration of total phenols is expressed as mg of GAE (gallic acid equivalents) per mL^−1^ of the sample.
- Antioxidant capacity (DPPH)
- The antioxidant capacity of the different beers was measured using the method described by Abderrahim et al. [23]. Beer samples, once filtered and diluted (the 50 μL sample or the blank control), were introduced and mixed with 1000 μL of DPPH (60 μMol L^−1^ dissolved in methanol 1: 1/10 mMol L^−1^ Tris-HCl buffer pH 7.5) in a 5 mL volumetric flask. At 0 min, and after 20 min of incubation at room temperature in the laboratory (21 ± 2 °C), a small volume was introduced into 10 mm quartz cuvettes, and absorbance was measured at 520 nm with the spectrophotometer mentioned above. The antioxidant capacity of the beer, expressed in μMol DPPH mL^−1^, was calculated using the following equation:
  µMol (DPPH mL^−1^) = ((A_0_ − At)/A_0_) × ((Vt [DPPH] × FD)/mL) (1)
  where A_0_: control absorbance (DPPH diluted in methanol); At: sample absorbance; Vt: total reaction volume in liters; [DPPH]: DPPH concentration; FD: dilution factor; and mL: sample milliliters used in the reaction.

### 2.6. Headspace Gas Chromatography–Mass Spectrometry Analysis (HS-GC–MS)

The preparation of the samples was carried out following the general method described by Liu and colleagues [24].

Samples were analyzed by HS/GC–MS (headspace gas chromatography coupled to a mass spectrometer) in a QP2010 Shimazdu device with an AOC 5000 autosampler and an HP-5MS column (30 m long, 0.25 mm internal diameter, and 25 μm of film).

Samples of 2 mL of each beer were previously filtered, placed in a 10 mL HS vial with NaCl 20% (*w*/*v*), and heated up to 80 °C at 250 rpm for 15 min; this was performed prior to the 100 ul HS injection of the sample in splitless mode.

Helium was used as a carrier gas, and the pressure was set at 110 kPa. The injector temperature was 120 °C, and the interface temperature was 250 °C. The oven followed the following program: an initial temperature of 40 °C for 2 min, a ramp-up of 10 °C/min to 140 °C, and a second ramp-up of 7 °C/min to 250 °C. Data were acquired in full scan mode in a m/z range of 30–350, and peak identification was made by comparison with the NIST08 and WILEY229 libraries.

### 2.7. Descriptive Sensory Analysis

#### 2.7.1. Panel of Judges

A panel of 8 professional beer tasters, 5 men and 3 women, was trained according to the ISO 8586:2012 standard at the premises of the company Cibus In para Agroalimentación S.L. Tasters were trained in the recognition of beer descriptors through six tasting sessions of 2 h each; they used a presence–absence scale and also rated quantification on a discontinuous 3-point scale. The first two sessions included identification training, using a free-choice profile technique and an identification test control. In the last four sessions, a tasting sheet was established that included the most important descriptors, using a focus group qualitative technique. The criteria for the possible elimination of a judge were considered and were specified for tasters who scored lower than 70% on the control identification test. Thus, a total of 2 tasters were eliminated, with 6 very well qualified tasters remaining.

#### 2.7.2. Sensory Evaluation Session

Sensory analysis was carried out during a single session at five o´clock in the afternoon. Before starting the evaluation session, tasters were informed of the project objective and the kinds of beers to be tasted. All beers were examined in duplicate; then, ten beers were served, with a break after the tasting of the first five. Sufficient amounts of each sample at 6 °C were served in glasses standardized according to ISO 3591:1977. Each sample was coded with random numbers of 3 digits; samples were served in a different order for each taster. Tests were carried out in a tasting room that met the recommended guidelines of the UNE-EN ISO 8589:2010. Tasters spent approximately two minutes with each sample, using a descriptive method with the same tasting sheet used in training, which contained descriptors that allowed them to characterize the beer sensory profile: visual (intensity, tonality, limpidity, froth color, and CO_2_ bubbles); aroma (maltiness, cereal malt, ripe fruit malt, hoppy, exotic fruit hop, citric fruit hop, herbaceous hop, yeast, tropical fruit yeast, spicy yeast, bread yeast, toasted, coffee, licorice, and caramel, as well as defects, such as: oxidized, cider, vinegar, musty, stable, and soapy); and taste (acidity, CO_2_, bitterness, body, and persistence). These descriptors comprised most of the classes and some of the first-tier terms reported in the beer sensory wheel.

### 2.8. Statistical Analysis

Statistical analysis was carried out using Xlstat v.2021.1 statistical software (Addinsoft, Paris, France).

Data analysis was performed to establish the differences between the averaged values for physicochemical measurements, total polyphenol content, and antioxidant capacity; analysis was conducted using an analysis of variance (one-factor ANOVA) and Tukey´s significant difference test (HSD), with statistical significance being set at a *p*-value < 0.05.

Principal component analysis (PCA) was used for HS-GC–MS, sensory analysis, and product characterization for the sensory data analysis.

## 3. Results and Discussion

### 3.1. Physicochemical Analysis

#### 3.1.1. Turbidity

White bread beers yielded similar values for turbidity compared to the control beer (see Table 3). With the exception of whole wheat bread beers, all beers made with bread presented lower values of turbidity than the control beer (ALE), with this result being statistically significant in the case of the beers made with corn bread and rye bread.

Whole wheat bread and white bread had the highest amounts of crumb, causing a higher content of suspended particles. On the other hand, the beer made with corn bread, as well as the one made with rye bread, had a very compact crumb, and the crust was much thicker, which favored the maceration process, releasing fewer solids into the wort and, therefore, generating less turbidity in the finished beers.

#### 3.1.2. Color (EBC Scale)

The results obtained herein show that the control beers were a darker color (a higher value on the EBC scale), while the beers brewed with bread exhibited paler colors. This may be because the bread dough (crumb and crust) added less EBC color value than toasted malt. Considering the bread-made beers, the bread crust color influenced the wort color and the finished beer darkness. Whole wheat bread was the darkest among the beers with added bread, due to its crust.

It can be concluded, after this analysis, that bread is capable of imparting color to beer, although to a lesser extent than malt.

#### 3.1.3. pH and Acidity

The control beers presented a pH range between 3.83 and 3.74, which was quite close to the expected value for these types of beers [25]. If we take this standard range as a reference, all beers made with bread showed similar pH values, without presenting significant differences between treatments with or without bread (Table 3).

As for acidity, no significant differences were observed in any of the treatments when making the 100% malt beer compared to the beers partially replacing malt with bread.

#### 3.1.4. Alcoholic Strength

With regard to alcoholic strength, it can be observed that the control beers reached a ABV of 4.33, a very similar value to that achieved by the beers made with white bread, whole wheat bread, and rye bread. This indicates that the sugars obtained from bread were as fermentable as those from malt; in addition, the lower enzyme content present in the wort, because of the 50% malt replacement with bread, did not affect the transformation into fermentable sugars, resulting in a similar alcoholic content. It must be noted, however, that there was a lower alcoholic strength achieved in the brewing of corn bread, which only attained a value of 3.39–3.49 ABV; this was a statistically significant difference from the other treatments.

#### 3.1.5. Dry Extract

The control beers were the ones with the highest dry extract values (5.71–5.53%); all bread beers presented lower values, especially the beers brewed with cornbread (4.31–3.96%), which presented the lowest values. This difference could be explained because the malt, in the absence of bread, was able to transfer more organic compounds into the wort during maceration.

### 3.2. Total Polyphenol Content (TPC) and Antioxidant Capacity in Craft Bread Beers

Phenolic compounds are generally considered one of the most important antioxidant sources in beer [26]. Phenolic compounds in beer are of great interest to brewers, as they directly affect beer quality. In addition to their positive effect on oxidation prevention, they can negatively influence colloidal and foam stability and, thus, shorten the shelf life of beer [25].

**Figure 1:**
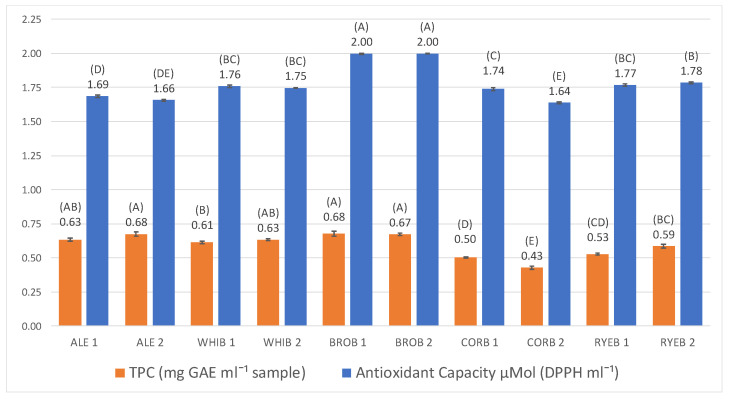
Antioxidant capacity and total polyphenol content in the craft bread beers studied. A–E means without any common letter within the same column are significantly different (*p* < 0.05).

Comparative results between beers with respect to TPC and antioxidant capacity are shown in Figure 1.

Figure 1 shows that beers made with whole wheat bread and white bread presented similar TPC values compared to control beers. Only in two treatments, namely those made with rye bread and corn bread, were TPC values lower and statistically significant compared to the others (Figure 1). The results obtained are in agreement with another study, in which four styles of craft beer were analyzed, and the total polyphenol content ranged from 448.57 to 531.30 mg GAE L^−1^ [27].

Regarding industrial beers, there are many references concerning TPC analysis; for instance, lager and pilsner beers ranged between 464 and 579 mg GAE L^−1^ [25]. In another research study, the TPC values of 34 lager beer samples were studied, and results ranged between 152.01 mg GAE L^−1^ and 339.12 mg GAE L^−1^ [28]. In another work, Indian pale lager beers were analyzed, obtaining values ranging from 160 to 620 mg GAE L^−1^ [29]. In addition, there are many studies that analyzed TPC in ale beers, such as wheat ale beers, which had a value of 403.1 mg GAE L^−1^ [30]; American pale ale beers, which showed a value of 540 mg GAE L^−1^ [31]; and blond ale beers, which had values ranging from 125 to 544.3 mg GAE L^−1^ [32]. In addition, there were two other works that investigated ale beers, obtaining values ranging from 382.7 to 563 mg GAE L^−1^ [33,34]. Finally, another research study examined dark ale beers, resulting in values ranging from 448.1 to 542.4 mg GAE L^−1^ [35]. It is worth mentioning that the choice of raw materials and the brewing processes used for different kinds of beers have a major influence on the polyphenol content of the final product [34].

All TPC values determined in this study (Figure 1) were in the same range as the cited references; higher values were found in the control beers, whole wheat beers, and white wheat beers, which also attained darker colors than the other bread beers. This positive correlation between TPC and EBC color was also found in other studies [30,34].

In addition, the results showed that beers brewed with corn bread had lower TPC values, which agrees with several studies carried out with beers brewed with corn malt [36,37].

With regard to the antioxidant capacity (Figure 1), beers made with whole wheat bread presented significantly higher levels of this parameter compared to the other brews studied, including the control beers. The results also showed that all beers made with bread, as a partial substitute for malt, had a higher antioxidant capacity than the control beer, except the corn bread beers. This is in accordance with a previous study of commercial beers [35], where somewhat lower values for antioxidant capacity were indicated, ranging from 0.56 to 1.66 μMol DPPH mL^−1^.

Replacing 50% of malt with bread increases the antioxidant capacity and, in general, the polyphenol content of beers, improving properties that would benefit the health of the consumers [38].

### 3.3. GC–MS Detection

The identification of the main volatile compounds present in beers was performed using library data, as described in Section 2.6. Relative quantification was performed as a percentage, relating the area under each peak to the sum of all peak areas. Thus, 14 volatile compounds were identified, but only 7 were included in the volatile profile due to their higher relative areas and discriminating power. The results for the seven main volatile compounds according to the retention times and peak area (%) are shown in Table 4.

**Figure 2:**
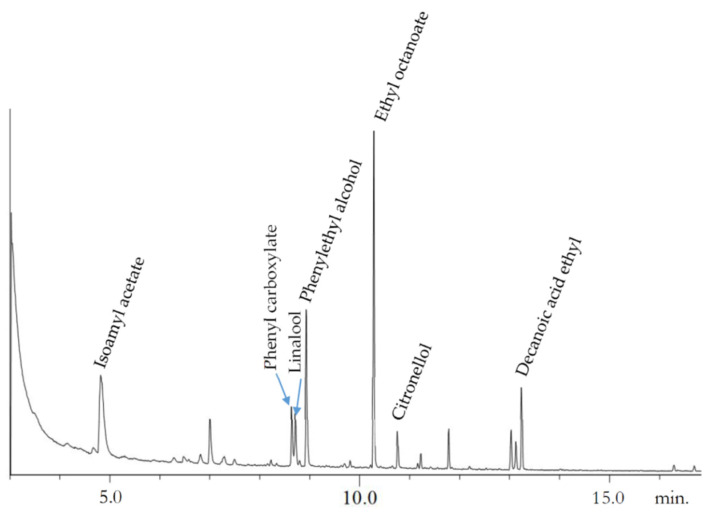
GC–MS chromatogram of whole wheat bread beer.

As an example, the chromatogram of the whole wheat bread beer is shown in Figure 2.

These main volatile compounds were also identified by other authors using the GC–MS technique [39,40,41]; those results showed high amounts of isoamylacetate, phenylethyl alcohol, and different ethyl compounds, plus other minority compounds, such as acid ethyl esters [41], linalool, and citronellol [39].

**Figure 3:**
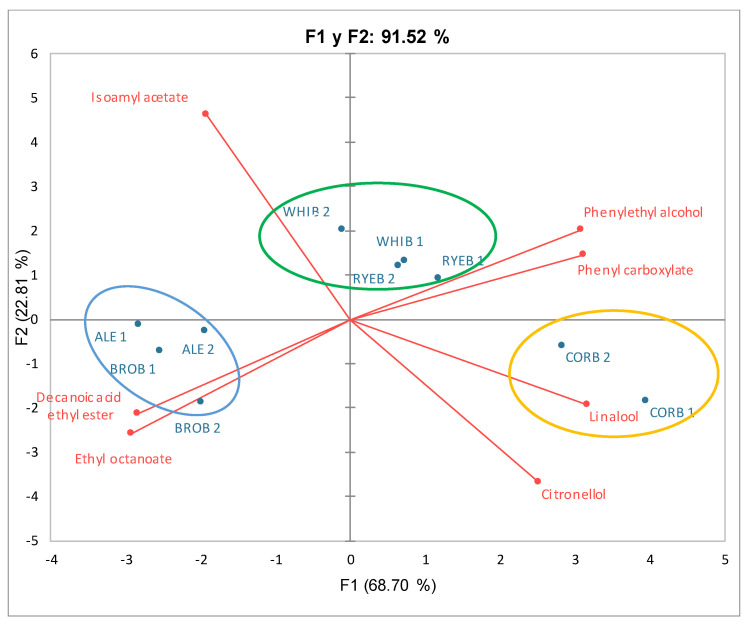
Principal component analysis of volatile compounds in analyzed bread beers.

PCA was applied to evaluate the data trends. Two principal components were extracted that explained 91.52% of the total variance. F1 explained up to 68.70% of the total variance, and F2 explained another 22.81%. By representing F1 versus F2, a scatter plot of the analyzed beers (biplot) was obtained (Figure 3). Representing our variables with respect to these two factors, it appears that the “isoamyl acetate” variable is totally independent of any other studied variables and is associated with a negative value for Factor 1 and a positive value for Factor 2.

Factor 1 had negative loadings for isoamyl acetate, ethyl octanoate, and decanoic acid ethyl ester, as well as high positive loadings for citronellol, linalool, phenylethyl alcohol, and phenyl carboxylate. Factor 2 had positive loadings for isoamyl acetate, phenylethyl alcohol, and phenyl carboxylate, as well as negative loadings for ethyl octanoate, decanoic acid ethyl ester, citronellol, and linalool.

Based on the results of the PCA and considering the studied beer samples, RYEB and WHIB were grouped together. This group of beers was the most neutral beers, which contained phenylethyl alcohol, phenyl carboxylate, and isoamyl acetate. On the other hand, BROB and ALE were also grouped together, and they appear in the lower left quadrant. This group of beers was characterized by the presence of ethyl octanoate and decanoic acid ethyl ester. Finally, in the lower right quadrant, the beer CORB was associated with the presence of citronellol and linalool (Figure 3).

### 3.4. Descriptive Sensory Analysis

#### 3.4.1. Visual and Taste Sensory Analysis

**Figure 4:**
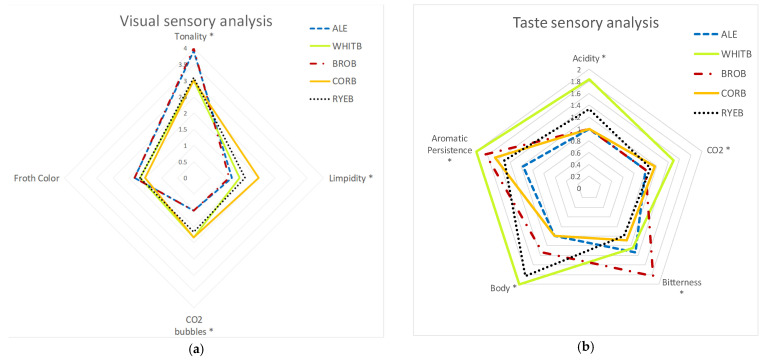
(**a**) Visual radar graphic; (**b**) Taste radar graphic. * Statistically significant differences (*p*-value ≤ 0.05).

The beer color analysis showed that the beer brewed with whole wheat bread attained the same color profile as the control beer, presenting a greater tonality; these results match those obtained experimentally for TPC (Figure 4a).

These results were in accordance with those of the physicochemical analysis, where control beers and whole wheat bread beers presented higher EBC color values and more turbidity than the other brewed beers.

Regarding the taste phase, all beers made with bread generally exhibited a profile with greater intensity in taste descriptors than the control beer. In particular, this was true for white wheat bread beer in terms of body, acidity, and CO_2_. In addition, the beer made with whole wheat bread presented greater bitterness, and this characteristic could be related to the increased content of polyphenols, providing the beer with a greater flavor intensity and longer persistence (Figure 4b).

The results obtained in the taste analysis show that the sensory profile varies considerably according to the beer analyzed. The beer brewed with white bread was the one that presented the highest values for acidity, CO_2_, body, and aromatic persistence.

On the other hand, the beer brewed with corn bread was the most similar to the control beer, with very similar values for acidity, body, and bitterness; however, this beer had a considerably higher aromatic persistence than the control beer (Figure 4b).

#### 3.4.2. Olfactory Sensory Analysis

**Figure 5:**
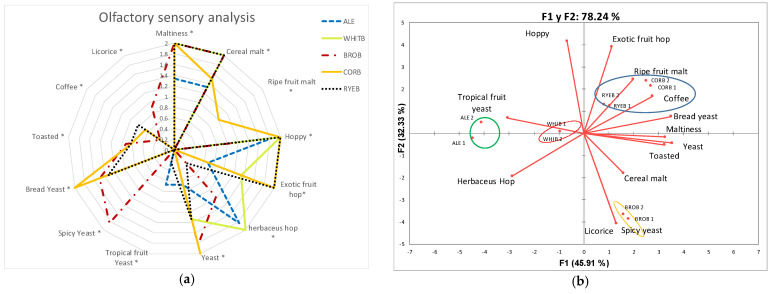
(**a**) Olfactory radar graphic; (**b**) PCA olfactory analysis. * Statistically significant differences (*p*-value ≤ 0.05).

Results from the olfactory descriptive analysis of the different beers showed that, in general, the beers brewed with bread had a more complex and intense odor profile than the control beer brewed with 100% malt (Figure 5a). However, all beers analyzed showed significant differences in terms of their main aromatic notes and some tasty flavors.

In order to corroborate the results obtained in the physicochemical analyses, a principal component analysis of the sensory variables was performed, obtaining two factors that explained 78.24% of the variance associated with the variables. In this PCA plot, F1 explained 45.91% of the total variance, and F2 explained another 32.33%. Representing our variables with respect to these two factors, it appears that the “herbaceous hop” variable was totally independent of the rest of the variables studied and was associated with negative values for Factor 1 and Factor 2 (Figure 5b).

Factor 1 had positive loadings for the majority of analyzed attributes: fruity exotic hop, ripe fruit malt, coffee, bread yeast, maltiness, yeast, toasted, cereal malt, spicy yeast, and licorice, as well as high negative loadings for hoppy, tropical fruit yeast, and herbaceous hop. Objects close together had similar characteristics; therefore, maltiness, yeast, and toasted variables appear to be highly positively correlated. Factor 2 had negative loadings for maltiness, yeast, toasted, cereal malt, spicy yeast, licorice, and herbaceous hop, as well as high positive loadings for fruity exotic hop, ripe fruit malt, coffee, bread yeast, fruity tropical yeast, and hoppy.

Based on the PCA results and considering the studied beer samples, CORB and RYEB were grouped together (Figure 5b). This group of beers showed exotic fruit hop, ripe fruit malt, coffee, and bread yeast flavors. In addition, the control beers were mainly associated with tropical fruit yeast flavor and were very close to beers brewed with white bread. Finally, in the lower right quadrant, the beer BROB was associated with the presence of licorice and spicy yeast flavors.

## 4. Conclusions

Brewing from bread is a viable alternative to traditional beer fermentation, although a significant fraction of malt is necessary. The percentage of malt that can be replaced by bread is up to 50%, meaning very important savings for the beer industry. All beers made by partially replacing malt with stale bread, except in the case of corn bread, achieved the same extraction of sugars, eventually reaching a similar alcoholic strength and physicochemical profile compared to the control beer, especially in the case of whole wheat bread beer.

Bread releases fewer particles into the brewing wort than malt, resulting in less cloudy and, in general, less intensely colored beers.

In particular, beers brewed with corn bread were of lower intensity, exhibiting lower values than the rest of the beers in terms of color, turbidity, alcoholic strength, dry extract, and total polyphenols; as such, corn bread beer can be defined as a lighter and weaker beer. In general, bread beers presented characteristic attributes in the different phases of the tasting, allowing them to be classified into different sensory profiles.

Beers brewed with whole wheat bread resulted in a product with physicochemical characteristics similar to that obtained using only malt, surpassing the rest of the elaborations made with bread. In addition, the total polyphenol content and antioxidant capacity make it a beer with healthier properties and better sensorial characteristics, providing a higher level of bitterness and greater persistence in the mouth. All of these results mean a great advancement and advantage for the brewing industry all over the world.

## Figures and Tables

**Table 1:** Main Brewing raw materials used in the elaboration of craft beers.

Grain Malt	Pilsen EBC 3 (Weyermann, Bamberg, Germany)
	Munich Type I EBC 12 (Weyermann, Bamberg, Germany)
	Cara Rye EBC 150 (Weyermann, Bamberg, Germany)
	Biscuit EBC 45 (Castle Malting, Verviers, Belgium)
Hops Pellets	Centennial 9.6% a.a. (Laguilhoat, Fuenlabrada, Spain)
	Cascade 6.7% a.a (Laguilhoat, Fuenlabrada, Spain)
	Simcoe 13.6% a.a. (Laguilhoat, Fuenlabrada, Spain)
Bread	White wheat bread
	Whole wheat bread
	Corn bread
	Rye bread
Yeast	SafAle S-04 (Fermentis, Marcq-en-Baroeul, France)
	SafAle F-2 (Fermentis, Marcq-en-Baroeul, France)
Water	Monte Pinos (Carbónicas Navalpotro S.A, Almazán, Spain)

**Table 2:** Characteristics of the brewing pelleted hops used, the hops’ addition time during boiling, and the amount of hops used in each treatment (5 L).

Variety of Hops	IBU	Alpha Acids (%)	Boil Min.	Weight (g)
Centennial	15	9.6	1	5.79
Cascade	10	6.7	30	7.86
Simcoe	5	13.6	59	3.34

**Table 3:** Values of the beer physicochemical properties (mean ± S.D.).

Beer/Analysis	Turbidity	Color (EBC)	pH	Acidity (% Lactic Acid)	ABV (%)	Dry Extract (%)
ALE 1	932.33 ± 29.57 ^C^	25.46 ± 0.03 ^A^	3.83 ± 0.06 ^ABC^	0.03 ± 0.01 ^A^	4.33 ± 0.06 ^AB^	5.71 ± 0.03 ^A^
ALE 2	975.67 ±15.63 ^B^	24.06 ± 0.01 ^B^	3.74 ± 0.01 ^CD^	0.03 ± 0.01 ^A^	4.33 ± 0.07 ^AB^	5.53 ± 0.11 ^AB^
WHIB 1	907.67 ± 8.33 ^C^	17.82 ± 0.04 ^E^	3.93 ± 0.01 ^A^	0.03 ± 0.01 ^A^	4.21 ± 0.07 ^B^	5.35 ± 0.05 ^BC^
WHIB 2	935.00 ± 3.61 ^C^	16.33 ± 0.04 ^F^	3.88 ± 0.04 ^AB^	0.03 ± 0.01 ^A^	4.37 ± 0.15 ^AB^	5.03 ± 0.05 ^DE^
BROB 1	1061.00 ± 3.46 ^A^	19.40 ± 0.11 ^C^	3.85 ± 0.04 ^ABC^	0.02 ± 0.01 ^A^	4.21 ± 0.10 ^B^	5.03 ± 0.08 ^DE^
BROB 2	1027.67 ± 6.51 ^A^	21.00 ± 0.21 ^D^	3.77 ± 0.01 ^BC^	0.03 ± 0.01 ^A^	4.58 ± 0.07 ^A^	4.94 ± 0.03 ^E^
CORB 1	743.67 ± 0.58 ^E^	11.74 ± 0.05 ^I^	3.81 ± 0.02 ^ABC^	0.02 ± 0.01 ^A^	3.39 ± 0.12 ^C^	3.96 ± 0.06 ^G^
CORB 2	689.67 ± 8.39 ^F^	11.75 ± 0.02 ^I^	3.85 ± 0.02 ^ABC^	0.02 ± 0.01 ^A^	3.49 ± 0.25 ^C^	4.31 ± 0.16 ^F^
RYE 1	834.67 ± 7.51 ^D^	13.24 ± 0.08 ^G^	3.64 ± 0.11 ^D^	0.02 ± 0.01 ^A^	4.25 ± 0.08 ^AB^	5.01 ± 0.03 ^DE^
RYE 2	708.00 ± 19.31 ^EF^	15.11 ± 0.10 ^H^	3.85 ± 0.02 ^ABC^	0.02± 0.01 ^A^	4.47 ± 0.06 ^AB^	5.23 ± 0.06 ^CD^

^A–G^ Means without any common letter within the same column are significantly different (*p* < 0.05).

**Table 4:** Comparison of retention times and peak areas (%) of volatile compositions in craft bread beers.

Beer/Compound	Isoamyl Acetate	Phenyl Carboxylate	Linalool	Phenylethyl Alcohol	Ethyl Octanoate	Citronellol	Decanoic Acid Ethyl Ester
Retention Time (min.)	4.83	8.64	8.72	8.94	10.28	10.75	13.24
ALE 1	32.45	3.49	4.59	18.61	12.05	2.40	9.09
ALE 2	28.39	5.33	4.64	21.28	15.08	2.26	5.97
WHIB 1	29.03	7.67	5.31	31.99	6.37	2.54	0.73
WHIB 2	33.33	7.69	5.20	26.98	6.46	1.87	0.89
BROB 1	25.94	3.69	4.17	15.35	16.39	2.05	4.12
BROB 2	23.50	5.07	5.19	14.03	18.58	2.97	5.60
CORN 1	18.20	8.63	9.72	33.87	3.41	5.25	0.37
CORN 2	21.40	10.29	8.29	30.86	5.71	3.79	0.71
RYE 1	28.84	7.80	6.55	28.78	4.40	2.93	0.54
RYE 2	28.11	8.46	5.94	27.43	6.52	2.08	0.62

## Data Availability

Data is contained within the article.
